# On the Stability of Steroids upon Gamma and E-Beam Irradiation and the Protective Effect of Inert Conditions

**DOI:** 10.3390/molecules30122605

**Published:** 2025-06-16

**Authors:** Quinten Speleers, Anke Meyers, Homaira Rashid, Yannick Dubbelboer, Elias Vanneste, Bart Croonenborghs, Annick Gillet, Aaron DeMent, Ann Van Schepdael, Erik Haghedooren

**Affiliations:** 1 Pharmaceutical Analysis, Department of Pharmaceutical and Pharmacological Sciences, KU Leuven—University of Leuven, Herestraat 49, O&N2, PB 923, 3000 Leuven, Belgium; 2 Sterigenics NV, a Sotera Health Division, 2015 Spring Road 650, Oak Brook, IL 60523, USA; bcroonenborghs@eu.sterigenics.com (B.C.); agillet@eu.sterigenics.com (A.G.); adement@sterigenics.com (A.D.); ehaghedooren@eu.sterigenics.com (E.H.)

**Keywords:** sterilization, irradiation, stability, ophthalmic, dexamethasone, methylprednisolone, inert conditions, dry conditions

## Abstract

The sterility of ophthalmic drugs is a fundamental requirement for ensuring patient safety, and as such, it is subject to stringent regulatory standards. However, significant gaps remain regarding the effect of sterilization techniques on the impurity profile and relative content of active pharmaceutical ingredients (API). Previous research involving a set of five APIs used in ophthalmic preparations (dexamethasone, methylprednisolone, aciclovir, tetracycline hydrochloride, and triamcinolone) demonstrated that gamma irradiation led to the formation of specific impurities in the corticosteroids, dexamethasone and methylprednisolone. This study aims to further explore the effect of both gamma and electron beam (E-beam) irradiation on the impurity profiles of these APIs under varying conditions, with and without dry ice. The analyses were conducted using high-performance liquid chromatography with ultraviolet/visible light (UV/VIS) detection and the effect of sterilization conditions was assessed in accordance with the assay and related substances test outlined in the European Pharmacopoeia (Ph. Eur.). Additionally, this study investigated whether exposure in a controlled atmosphere with reduced oxygen or water content could mitigate the formation of impurities and influence the stability of the compounds. The results indicated a protective effect of low-temperature and low-oxygen environments during both gamma and E-beam irradiation but no effect of dry conditions.

## 1. Introduction

In the healthcare sector, the production of high-quality products that are safe to use is paramount [1]. To ensure the quality, safety, and efficacy of particular types of pharmaceutical products, manufacturing processes are conducted under aseptic conditions or sterility is obtained through terminal sterilization.

Terminal sterilization is preferred over aseptic processing as the product undergoes sterilization in the final container closure system, avoiding the risk of contamination during aseptic processing and hence providing the greatest assurance of sterility [2,3]. Three main categories of terminal sterilization exist: thermal sterilization (dry heat or steam), ionizing radiation sterilization (X-rays, gamma rays, or electron beam [E-beam]), and gas sterilization (e.g., ethylene oxide [EO], nitrogen dioxide [NO_2_], or vaporized hydrogen peroxide [VH_2_O_2_]) [3,4].

A sterilization process could cause degradation of the pharmaceutical preparation, resulting in a lower content and/or an increase in impurities. Acceptable limits of impurities provided by the European Pharmacopoeia (Ph. Eur.) should be respected, as impurities above those limits could impose a health risk for patients. As many drugs cannot withstand the high temperatures of the thermal sterilization methods, ionizing radiation sterilization is often used as an alternative [5,6]. The present study therefore focuses on the effect of both gamma and E-beam irradiation on five different ophthalmic active pharmaceutical ingredients (APIs) (dexamethasone, methylprednisolone, aciclovir, tetracycline hydrochloride, and triamcinolone).

Gamma irradiation and E-beam irradiation use Cobalt-60 (^60^Co) gamma rays and accelerated electrons as sterilizing agent, respectively. For both types of radiation, ionization events will damage or break essential biomolecules inside microorganisms [7]. Compared with ^60^Co gamma rays, the penetration capabilities of an E-beam are substantially more limited [6,8].

The literature about the effect of gamma irradiation on the ophthalmic APIs studied in this paper remains rather limited. A study investigating the effects of gamma irradiation on corticosteroids, including methylprednisolone, identified two major degradation pathways: the loss of the corticoid side chain to produce the C-17 ketone and the oxidation of the C-11 alcohol [9]. Most of the included corticosteroids were considered stable upon exposure to gamma irradiation. Another study identified three major degradation pathways following gamma irradiation of dexamethasone: ·OH radical oxidation, ·OH radical substitution, and the direct decomposition of dexamethasone [10]. Gamma irradiation of antibiotics, including tetracycline, generally does not result in discernible degradation, except for a minor change in tint [11]. One article showed that there was no effect of irradiation on tetracycline powder after exposure to a dose within the range of 25 kGy to 80 kGy. Aqueous solutions, however, were already severely affected at a dose of 25 kGy [12]. One study found that aciclovir microspheres maintained their stability after being exposed to a dose of 25 kGy [13].

A recent study investigated the effects of both EO and gamma sterilization under non-inert and non-dried conditions on the content and impurity profile of dexamethasone, methylprednisolone, aciclovir, tetracycline hydrochloride, and triamcinolone [14]. EO sterilization of dexamethasone and methylprednisolone did not result in significant impurities. Some impurities were observed following EO sterilization of aciclovir, tetracycline hydrochloride, and triamcinolone, but these complied with the limits outlined in the Ph. Eur. Gamma irradiation of dexamethasone and methylprednisolone did result in a notable increase in certain impurities. This included the formation of impurity C, resulting from the dealkylation of methylprednisolone at the C-17 position. Under the specified irradiation conditions, the other APIs complied with the limits outlined in the Ph. Eur. [14].

The literature about the effect of E-beam irradiation on the ophthalmic APIs studied in this paper is even more scarce. A study investigating the effect of E-beam irradiation on steroids showed that E-beam influenced the C-11 alcohol, which forms a carbonyl after irradiation [6,15].

The sterilization of products by ionizing radiation in the presence of oxygen or water generates free radicals, which may cause secondary degradation. To evaluate the effect of oxygen and water on both the impurity profile and the relative content of the API, the working atmosphere can be changed. An atmosphere with a lower oxygen content, i.e., an inert atmosphere, can be obtained either by replacing the oxygen with a non-oxidative gas (e.g., nitrogen, carbon dioxide, or argon) or by removing the oxygen with a vacuum [16]. An API with a lower water content can be created by drying the API in an oven until a stable weight is reached.

The current study was designed to examine the behavior of the ophthalmic APIs as drug substances, mainly focusing on the steroids (dexamethasone and methylprednisolone) under gamma and E-beam irradiation with various absorbed doses, at ambient temperature conditions and in the presence of dry ice to cool the sample down during processing at the sterilization site. It was also investigated whether a treatment atmosphere with lower oxygen content (inert conditions) or lower water content (dry conditions) would improve the behavior of the compounds and decrease the generation of impurities. The aim was to verify whether the compounds would still comply with an official compendium after the irradiation treatments. Hence, the effect of the sterilization methods on the relative content and the related substances was assessed using high performance liquid chromatography (HPLC) coupled to an ultraviolet/visible light (UV/VIS) detector following the procedures mentioned in the Ph. Eur. monographs. This implies that all impurities above the disregard limit were monitored.

## 2. Results and Discussion

### 2.1. Gamma and E-Beam Irradiation Under Non-Inert and Non-Dried Conditions

#### 2.1.1. Dexamethasone

##### Related Substances

**Figure 1:**
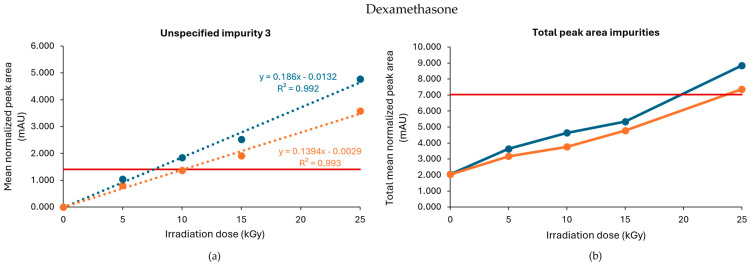
Effect of gamma irradiation on impurities in dexamethasone samples under non-inert and non-dried conditions. The graphs present the correlation between the irradiation dose (x-axis) and (**a**) the mean normalized peak area of unspecified impurity 3 and (**b**) the sum of the mean normalized peak area of all the impurities above the disregard limit (y-axis) after exposure of dexamethasone samples to gamma irradiation under those conditions, processed with or without dry ice. Blue line: samples processed without dry ice; Orange line: samples processed with dry ice; Red line: limit specified in the monograph [17]. The non-treated sample stored in the lab is represented as the 0 kGy sample. The limits per dose are slightly different since they are sample-dependent and, therefore, the average limit for both conditions has been shown here. All peak areas were normalized for mass. Standard deviations are plotted for each data point (all standard deviations [SDs] are ≤0.05 mAU).

**Figure 2:**
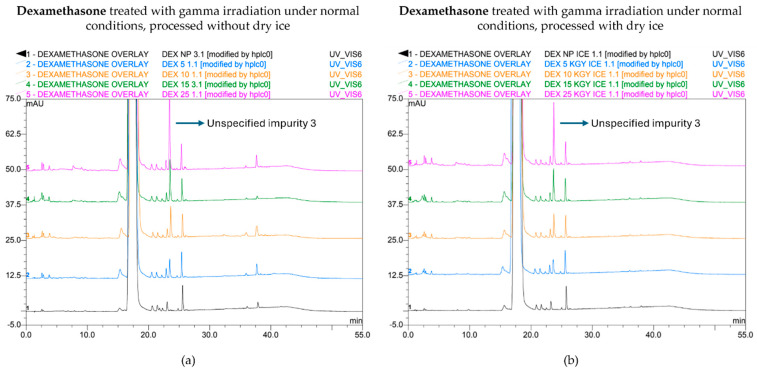
Chromatogram overlay showing the impurity profiles of dexamethasone for all irradiation doses, when treated with gamma rays under non-inert and non-dried conditions and processed without (**a**) and with (**b**) dry ice. From bottom to top: non-processed sample (0 kGy), sample exposed to 5 kGy, 10 kGy, 15 kGy, and 25 kGy.

When processed without dry ice, unspecified impurity 3 exceeded the limit when the samples were treated with gamma irradiation at 10, 15, and 25 kGy, while the limit was only exceeded when treated with 15 and 25 kGy when the dexamethasone samples were processed with dry ice (Figure 1a). Of note, an unspecified impurity is defined by Ph. Eur. as an impurity that is limited by a general acceptance criterion and not individually listed with its own acceptance criterion. On the other hand, the limit of the total peak area for all impurities, calculated as the sum of every mean normalized peak area that exceeded the disregard limit, was surpassed in both conditions (with or without dry ice) when treated with a dose of 25 kGy (Figure 1b). Overall, higher peak areas for both unspecified impurity 3 and total impurities were found for each irradiation dose when dexamethasone samples were processed without dry ice compared with those processed with dry ice. The corresponding chromatograms are shown in Figure 2.

To determine a maximum irradiation dose for unspecified impurity 3, to stay within the prescribed limit set by the Ph. Eur., a linear regression curve was estimated for all conditions. The estimated maximum acceptable irradiation dose was 8 kGy when the samples were processed without dry ice and 10 kGy when the samples were processed with dry ice (Table 1, Figure 1). In comparison, Van Cauwenbergh et al. showed that after gamma irradiation under non-inert and non-dried conditions, with doses up to 25 kGy, unspecified impurity 3 and the total peak area of all impurities were increased above the limit specified in the monograph [17], both in samples processed with or without dry ice [14].

**Figure 3:**
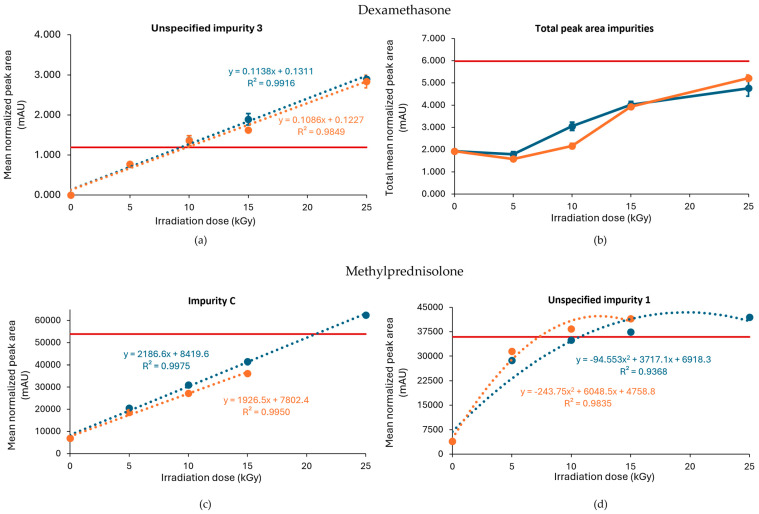
Effect of E-beam irradiation dose on impurities in dexamethasone and methylprednisolone samples under non-inert and non-dried conditions. Graphs present the correlation between the irradiation dose and (**a**) unspecified impurity 3, (**b**) the total peak area of all impurities after exposure of dexamethasone, (**c**) impurity C (11β-hydroxy-6α-methylandrosta-1,4-diene-3,17-dione), and (**d**) unspecified impurity 1 after exposure of methylprednisolone to E-beam irradiation under those conditions, processed with or without dry ice. Blue line: samples processed without dry ice; Orange line: samples processed with dry ice; Red line: limit specified in the monograph [17,18]. The non-treated sample stored in the lab is represented as the 0 kGy sample. The limits per dose are slightly different since they are sample dependent and, therefore, the average limit for both conditions has been shown here. All peak areas were normalized for mass. Standard deviations are plotted for each data point.

**Figure 4:**
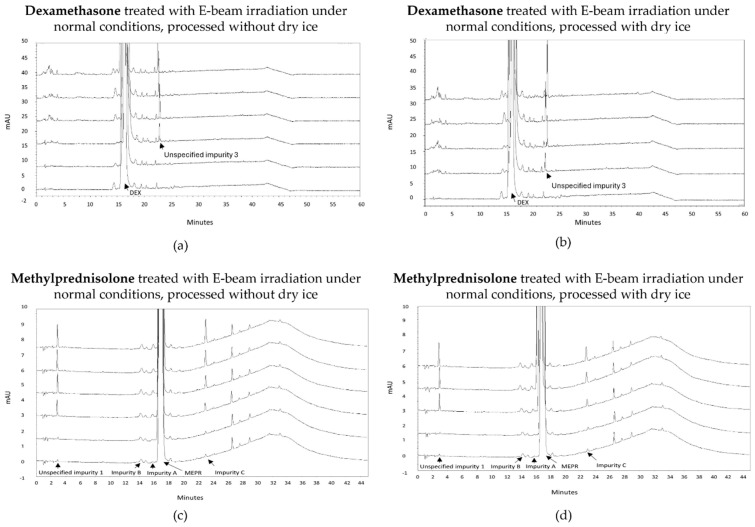
Chromatogram overlay showing the impurity profiles of dexamethasone (**a**,**b**) and methylprednisolone (**c**,**d**) for all irradiation doses, when treated with E-beam irradiation under non-inert and non-dried conditions and processed without (**a**,**c**) and with (**b**,**d**) dry ice. From bottom to top for both dexamethasone and methylprednisolone: non-processed sample (0 kGy), sample exposed to 5 kGy, 10 kGy, 15 kGy, and 25 kGy.

An increase in impurities was also observed after E-beam treatment of dexamethasone samples under non-inert and non-dried conditions (Figure 3a,b). It may be noted that the applied range of E-beam doses for dexamethasone and the other APIs was chosen to match those of previous experiments with gamma irradiation [14]. Unspecified impurity 3 exceeded the acceptable limits at irradiation doses of 10, 15, and 25 kGy for both samples processed with and without dry ice. On the other hand, the sum of the mean normalized peak areas of all impurities above the disregard limit did not exceed the acceptable limit up to the greatest dose in the study but still increased with an increasing irradiation dose. The corresponding chromatograms are shown in Figure 4a,b.

Based on the linear regression curve of the mean normalized peak area of unspecified impurity 3 in function of the irradiation dose, an estimation could be made of the maximum irradiation dose. The estimated maximum acceptable dose was for both conditions 9 kGy (Table 1, Figure 3a,b).

##### Relative Content

To determine the content of the treated dexamethasone samples, test solutions were prepared as described in the monograph (dissolve 25.0 mg of substance in 1.5 mL of acetonitrile, add 5 mL of mobile phase A, sonicate, and dilute to 10.0 mL with mobile phase A) [17]. No substantial decrease or increase was observed for the relative content of the dexamethasone samples after gamma irradiation under non-inert and non-dried conditions, both processed with or without dry ice, as the relative contents varied between 97.1% and 101.6%. Similarly, E-beam irradiation under those conditions did not cause manifest changes in relative contents of the dexamethasone samples, as the relative contents ranged from 98.7% to 102.9% (Table 2).

#### 2.1.2. Methylprednisolone

##### Related Substances

As described in a previously published paper by Van Cauwenbergh et al., after the gamma irradiation of methylprednisolone samples under non-inert and non-dried conditions, a positive correlation was observed between the irradiation dose and the presence of unspecified impurity 1 as well as impurity C (11β-hydroxy-6α-methylandrosta-1,4-diene-3,17-dione). Unspecified impurity 1 exceeded the limit for every irradiation dose tested in that study (15, 25, and 35 kGy) [14].

Similar to gamma irradiation, the E-beam treatment of methylprednisolone samples under non-inert and non-dried conditions resulted in an increase in several impurities with increasing irradiation doses, namely unspecified impurity 1 and impurity C (Figure 3c,d), as well as impurity A (17,21-dihydroxy-6α-methylpregna-1,4-diene-3,11,20-trione) and impurity B (11β,17,21,21-tetrahydroxy-6α-methylpregna-1,4-diene-3,20-dione). The mean normalized peak areas of impurity A and impurity B both still complied with the limits prescribed in the monograph [18]. For the samples processed without dry ice, unspecified impurity 1 did not comply with the limits stated in the monograph [18], when subjected to an irradiation dose of 15 and 25 kGy, and impurity C did not comply with the Ph. Eur. limits, when subjected to 25 kGy. The increase in impurity C is the result of a cleavage between C-17 and C-20 [9]. In addition, methylprednisolone samples processed with dry ice did not meet the limits for unspecified impurity 1 upon irradiation doses of 10 and 15 kGy. Overall, an increase in total peak area was observed with an increasing irradiation dose. The corresponding chromatograms are shown in Figure 4c,d. To make an estimation of the maximum acceptable E-beam dose, linear regression was performed for impurity C with R^2^ values of 0.995 and 0.998 for the samples processed with and without dry ice, respectively. The linear regression of unspecified impurity 1 resulted in low R^2^ values of 0.814 and 0.709, respectively. Therefore, quadratic regression was performed for this impurity resulting in R^2^ values of 0.984 for the samples processed with dry ice and 0.937 for the samples processed without dry ice. Based on the linear regression of impurity C and the quadratic regression of unspecified impurity 1, the estimated maximum acceptable E-beam dose was 7 kGy for the samples processed with dry ice and 10 kGy for the samples processed without dry ice (Table 1, Figure 3c,d).

##### Relative Content

To determine the content of the treated methylprednisolone samples, test solutions were prepared as described in the monograph (dissolve 30.0 mg of substance in phosphoric acid–acetonitrile–water (0.1:50:50, *V*/*V*/*V*) and dilute to 50.0 mL with this mixture.) [18]. Gamma irradiation under non-inert and non-dried conditions did not cause manifest changes in relative contents of methylprednisolone samples [14]. In addition, E-beam irradiation under those conditions of methylprednisolone samples did not cause a profound change in relative content, as the relative content ranged from 99.0% to 100.9% (Table 2).

It can be concluded from the above that both steroids were affected by gamma or E-beam treatment under non-inert and non-dried conditions. This is why these compounds were also irradiated under inert and dried conditions (see sections below) in order to investigate whether the irradiation effect could be attenuated.

The results obtained for the other three ophthalmic APIs tetracycline hydrochloride, aciclovir, and triamcinolone, treated with E-beam irradiation under non-inert and non-dried conditions are described in the next Section 2.1.3.

#### 2.1.3. Tetracycline Hydrochloride, Aciclovir, and Triamcinolone

A description of the behavior of these compounds after gamma irradiation under non-inert and non-dried conditions can be found in [14].

##### Related Substances

After the exposure of tetracycline hydrochloride samples to E-beam irradiation under non-inert and non-dried conditions, no major increases were observed in the mean normalized peak areas of four predetermined impurities, i.e., impurity A, impurity B, impurity C, and impurity D. For each irradiation dose and for each condition (samples processed with or without dry ice), the levels of these impurities were below their individual limits as stated in the monograph [19], which is consistent with the findings when tetracycline hydrochloride was exposed to gamma irradiation under similar conditions reported in earlier studies [14,20]. Impurity A did show small fluctuations in its normalized peak areas across the various irradiation doses, while the levels of the other impurities remained relatively stable. These fluctuations of impurity A, which is also called 4-epitetracycline, could possibly be explained by the reversible epimerization between tetracycline and 4-epitracycline. Overall, no beneficial effects were observed when delivering the irradiation dose in two passes compared with delivering it in one single pass (Table 3).

For aciclovir, a small increase in the mean normalized peak areas of both impurity B and impurity P with increasing irradiation dose was shown after exposure to E-beam irradiation under non-inert and non-dried conditions, both in samples processed with and without dry ice. For each irradiation dose, the levels of these impurities were below their individual limits as stated in the monograph [21]. These results of E-beam irradiation under non-inert and non-dried conditions align with the effect of gamma irradiation on aciclovir that was found in a previous study [14]. Overall, and similar to tetracycline hydrochloride, no beneficial effects were observed when delivering the irradiation dose in two passes compared with delivering it in one single pass (Table 4).

**Figure 5:**
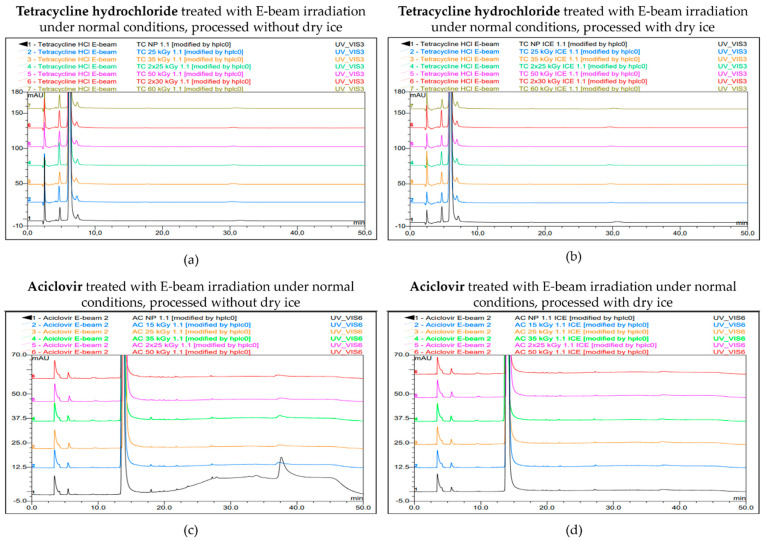
Chromatogram overlay showing the impurity profiles of tetracycline hydrochloride (**a**,**b**) and aciclovir (**c**,**d**) for all irradiation doses, when treated with E-beam irradiation under non-inert and non-dried conditions and processed without (**a**,**c**) and with (**b**,**d**) dry ice. From bottom to top for tetracycline hydrochloride: non-processed sample (0 kGy), sample exposed to 25 kGy, 35 kGy, 2 × 25 kGy, 50 kGy, 2 × 30 kGy, and 60 kGy. From bottom to top for aciclovir: non-processed sample (0 kGy), sample exposed to 15 kGy, 25 kGy, 35 kGy, 2 × 25 kGy, and 50 kGy.

The chromatograms for both tetracycline hydrochloride and aciclovir are shown in Figure 5.

For all the doses delivered under non-inert and non-dried conditions to the triamcinolone samples (up to 60 kGy; in one or two passes, both with and without dry ice), the compound still complied with the related substance specifications of the triamcinolone monograph (0.5% limit for any impurity and 2.0% limit for total impurities) [22]. These results are in line with the gamma irradiation of triamcinolone described before [14].

##### Relative Content

To determine the content of the treated tetracycline hydrochloride samples, test solutions with a concentration of 1.0 mg/mL were prepared in 0.01 M hydrochloric acid as described in the monograph [19]. No major changes in the content relative to the non-processed sample were observed after E-beam irradiation, both for the samples processed with and without dry ice. The relative contents varied between 97.7% and 101.5%. All the obtained *p*-values exceeded α, so no significant differences in content between the samples were found (Table 5).

Test solutions with a concentration of 1.0 mg/mL were prepared as described in the monograph (dissolve 25 mg of substance in 5.0 mL of dimethyl sulfoxide and dilute to 25.0 mL with water) and then tenfold diluted to determine the content of the treated aciclovir samples [21]. No major changes were obtained in their contents relative to the non-processed sample, as the range of the relative contents was between 98.6% and 102.6%. Only a significant difference between the non-processed sample and the sample sterilized with 25 kGy under non-inert and non-dried conditions processed without dry ice was found, as it resulted in an increased mean normalized peak area with a *p*-value of 0.016. However, this significant finding can be a result of the small values of the RSDs or can be assigned to possible analytical errors (Table 5).

The relative content for the triamcinolone samples irradiated with E-beam is shown in Table 5.

### 2.2. Gamma and E-Beam Irradiation Under Inert Conditions

#### 2.2.1. Dexamethasone

##### Related Substances

**Figure 6:**
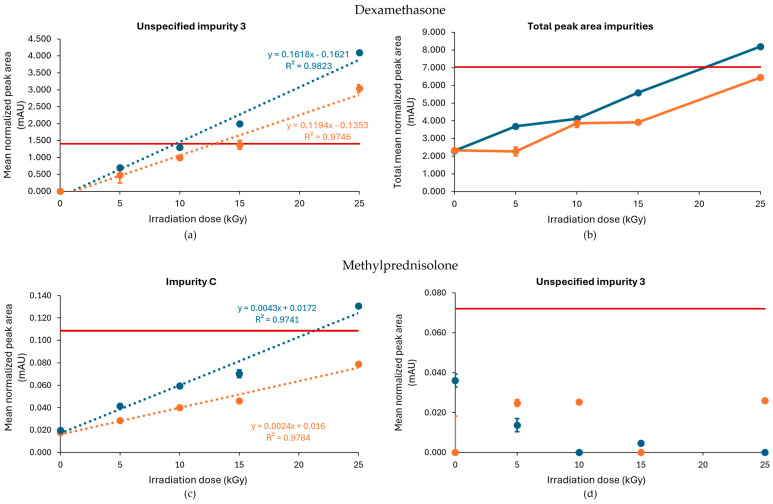
Effect of gamma irradiation dose on impurities in dexamethasone and methylprednisolone samples under inert conditions. Graphs present the correlation between the irradiation dose and (**a**) unspecified impurity 3, (**b**) the total peak area of all impurities after exposure of dexamethasone, (**c**) impurity C, and (**d**) unspecified impurity 1 after exposure of methylprednisolone to gamma irradiation under inert conditions, processed with or without dry ice. Blue line: samples processed without dry ice; Orange line: samples processed with dry ice; Red line: limit specified in the monograph [17,18]. The non-treated sample stored in the lab is represented as the 0 kGy sample. The limits per dose are slightly different since they are sample dependent and, therefore, the average limit for both conditions has been shown here. All peak areas were normalized for mass. Standard deviations are plotted for each data point.

**Figure 7:**
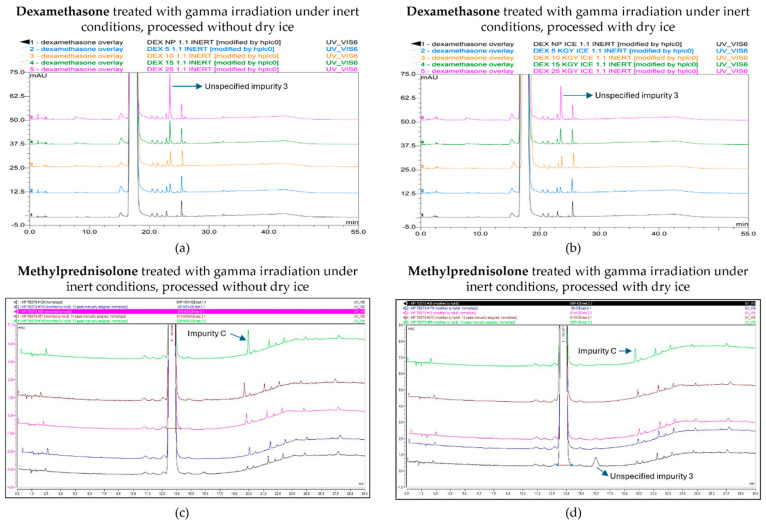
Chromatogram overlay showing the impurity profiles of dexamethasone (**a**,**b**) and methylprednisolone (**c**,**d**) for all irradiation doses, when treated with gamma rays under inert conditions and processed without (**a**,**c**) and with (**b**,**d**) dry ice. From bottom to top for both dexamethasone and methylprednisolone: non-processed sample (0 kGy), sample exposed to 5 kGy, 10 kGy, 15 kGy, and 25 kGy.

Following the gamma treatment of dexamethasone under an inert atmosphere, higher irradiation doses increased the total number of impurities as well as unspecified impurity 3 in a dose-dependent manner, and both exceeded the limit stated in the monograph (Figure 6a,b) [17]. Unspecified impurity 3 exceeded the limit for the first time at a dose of 15 kGy in samples processed without dry ice, while this only occurred at the highest dose of 25 kGy when samples were processed with dry ice. Overall, the peak area of unspecified impurity 3 was higher at all irradiation doses for samples processed without dry ice. The total peak area of all impurities showed higher levels for samples processed without dry ice compared with samples processed with dry ice, with the 25 kGy dose being the only condition that did not comply with the prespecified limit in samples processed without dry ice. The corresponding chromatograms are shown in Figure 7a,b.

Based on the linear regression curve of the mean normalized peak area of unspecified impurity 3 as a function of the irradiation dose, with R^2^ values of 0.975 and 0.982 for the samples processed with and without dry ice, respectively, an estimation could be made of the maximum acceptable irradiation dose. This estimate was 13 kGy for samples processed with dry ice and 10 kGy for samples processed without dry ice (Table 1, Figure 6a).

**Figure 8:**
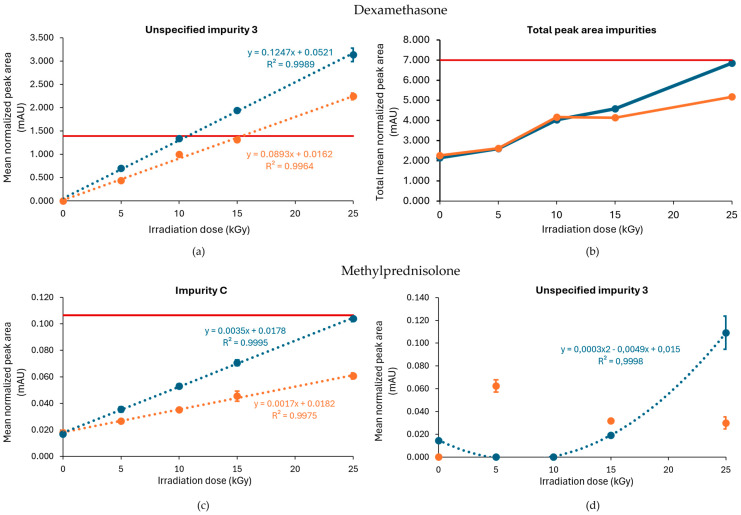
Effect of E-beam irradiation dose on impurities in dexamethasone and methylprednisolone samples under inert conditions. Graphs present the correlation between the irradiation dose and (**a**) unspecified impurity 3, (**b**) the total peak area of all impurities after exposure of dexamethasone, (**c**) impurity C, and (**d**) unspecified impurity 3 after exposure of methylprednisolone to E-beam irradiation under inert conditions, processed with or without dry ice. Blue line: samples processed without dry ice; Orange line: samples processed with dry ice; Red line: limit specified in the monograph [17,18]. The non-treated sample stored in the lab is represented as the 0 kGy sample. The limits per dose are slightly different since they are sample dependent and, therefore, the average limit for both conditions has been shown here. All peak areas were normalized for mass. Standard deviations are plotted for each data point, except for graph (**b**) in which each data point represents a sum of several mean normalized peak areas.

The E-beam irradiation of dexamethasone under inert conditions showed a similar effect of dry ice processing on the impurity profile as reported for gamma irradiation, with a higher level of impurities detected in samples processed without dry ice. In contrast to gamma irradiation, the total peak area of impurities remained below the Ph. Eur. limit (Figure 8b). Specific impurities that accumulated with increasing irradiation doses included unspecified impurity 3, impurity J (17,21-dihydroxy-16α-methylpregna-1,4-diene-3,11,20-trione), and, to a lesser extent, unspecified impurity 1. From those, only unspecified impurity 3 surpassed the Ph. Eur. limit at a dose of 25 kGy and 15 kGy in samples processed with and without dry ice, respectively. With R^2^ values of 0.996 and 0.998 for the samples processed with and without dry ice, respectively, an estimation could be made of the maximum acceptable irradiation dose for unspecified impurity 3. The theoretical maximum acceptable radiation dose was 15 kGy for samples processed with dry ice and 10 kGy for samples processed without dry ice (Table 1). The slope of the regression equation when the samples were processed without dry ice (0.1247) was larger than the one when the samples were processed with dry ice (0.0893), which indicates that unspecified impurity 3 increases more rapidly when irradiated in the absence of dry ice (Figure 8a).

##### Relative Content

Gamma irradiation under inert conditions did not substantially affect the content of dexamethasone relative to the non-processed sample, irrespective of processing with or without dry ice. The relative contents varied between 97.4% and 102.4%, with two samples showing significance at the 95% confidence level. These significant *p*-values are potentially due to analytical errors, small RSDs, and/or the influence of light during preparation, which was prevented as much as possible by the use of aluminium foil to cover the glassware during the preparation and the use of brown HPLC vials. Similar results were observed for E-beam irradiation, as the relative contents varied between 97.9% and 103.7%. The inert conditions did not induce major changes in the relative content of dexamethasone, except for the 10 kGy irradiation dose, which significantly increased the relative content in samples processed with and without dry ice with a *p*-value of 0.021. Although both irradiation methods showed a few significant differences in relative dexamethasone content, we expect those are mainly related to technical errors and small RSD values (Table 6).

#### 2.2.2. Methylprednisolone

##### Related Substances

As described above, previous studies found a positive correlation between the irradiation dose and the presence of unspecified impurity 1 and impurity C after the gamma irradiation of methylprednisolone samples under non-inert and non-dried conditions [14]. However, under inert conditions, the level of unspecified impurity 1 was lower. While this condition did increase the amount of impurity C proportional to dose—which was more pronounced in samples stored without dry ice, where the amount surpassed the limit at 25 kGy—the increase was lower compared with non-inert and non-dried conditions, without the detection of any other impurities that crossed the Ph. Eur. limit [18]. Furthermore, the non-processed sample with dry ice storage also showed an increase in impurity 3, but this was attributed to a technical error and can be ignored. The corresponding chromatograms are shown in Figure 7c,d. Based on the linear regression curve of the mean normalized peak area of impurity C in function of the irradiation dose, with R^2^ values of 0.978 and 0.974 for the samples processed with and without dry ice, respectively, an estimation could be made of the maximum acceptable irradiation dose (Figure 7c). As a result, it is advisable to not exceed a gamma irradiation dose for inert conditions without dry ice of 21 kGy (Table 1). Analysis of these linear regression curves showed that the slope for samples processed without dry ice (0.0043) was higher compared with the slope for samples processed with dry ice (0.0024), indicating that impurity C increased more rapidly with the used irradiation dose in conditions without the use of dry ice (Figure 7c).

**Figure 9:**
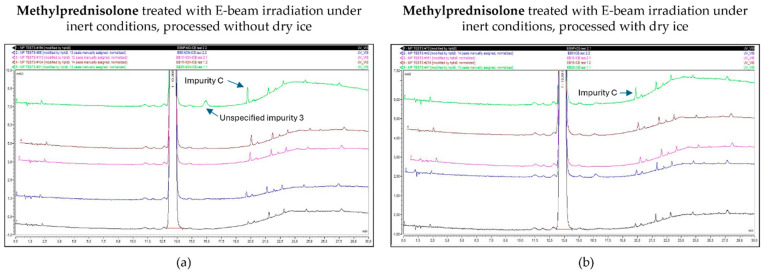
Chromatogram overlay showing the impurity profiles of methylprednisolone for all irradiation doses, when treated with E-beam irradiation under inert conditions and processed without (**a**) and with (**b**) dry ice. From bottom to top: non-processed sample (0 kGy), sample exposed to 5 kGy, 10 kGy, 15 kGy, and 25 kGy.

As described above, the E-beam irradiation of methylprednisolone samples under non-inert and non-dried conditions resulted in an increase in impurities, especially unspecified impurity 1 and impurity C. After the inertization of methylprednisolone, the results improved. The impurities that approached or exceeded the Ph. Eur. limit during E-beam irradiation in inert conditions were limited to impurity C and unspecified impurity 3, each at a dose of 25 kGy. However, only samples processed without dry ice showed this pattern, while all the detected impurities in samples processed with dry ice remained within the appropriate range. The corresponding chromatograms are shown in Figure 9.

Dose linear regression was calculated under the inert conditions for both impurity C and unspecified impurity 3. This resulted in R^2^ = 0.998 for impurity C for conditions with dry ice and R^2^ = 0.999 for conditions without dry ice (Figure 8c). For unspecified impurity 3, a quadratic regression was performed for the condition without dry ice. With a R^2^ = 0.999, the maximum acceptable irradiation dose for inert conditions without dry ice could be estimated as 24 kGy (Table 1, Figure 8d).

##### Relative Content

The relative content of methylprednisolone after either gamma or E-beam irradiation under inert conditions showed significant changes relative to the non-processed sample, with a consistent increase for all samples processed with dry ice, without any clear relationship to dose or irradiation method. The relative contents ranged from 95.3% to 108.0% for inert samples exposed to gamma irradiation and 93.2% to 106.0% for inert samples exposed to E-beam irradiation. The underlying mechanism for this increase is still unknown. Samples processed without dry ice illustrated a decline in relative content, but this occurred randomly rather than proportional to dose (Table 6).

### 2.3. Gamma and E-Beam Irradiation Under Dried Conditions

Dexamethasone and methylprednisolone samples were dried for 3 h at 105 °C to decrease the water content of the samples in an attempt to lower the formation of hydroxyl radicals that form during irradiation. The drying itself resulted in APIs that still complied with the respective monographs. However, some related substances such as impurities C and D in methylprednisolone had increased in peak area. Other drying conditions were investigated, but no efficient conditions could be found that maintained the original impurity profile of the compounds (drying to constant mass at 105 °C induced degradation of the compounds and drying at room temperature in a desiccator over silica (20 h) or phosphorus pentoxide (24 h) did not induce a mass loss).

Hence, gamma and E-beam irradiations were performed at a 25 kGy dose on these 3 h-dried dexamethasone and methylprednisolone samples, and the results are shown in Table 7. Overall, the various impurities behaved the same or worse than reported above for the other irradiations; therefore, it can be concluded from Table 7 that prior drying did not have a protective effect during the irradiation.

## 3. Materials and Methods

### 3.1. Active Pharmaceutical Ingredients

The five investigated APIs (i.e., dexamethasone, methylprednisolone, tetracycline hydrochloride, aciclovir, and triamcinolone) were all obtained from Fagron (Nazareth, Belgium). The European Directorate for the Quality of Medicines and Healthcare (EDQM, Strasbourg, France) has provided all the chemical reference substances that were used to identify the peaks on the chromatograms due to the various impurities.

### 3.2. Chemicals

Acetonitrile 99.9% for HPLC gradient grade, methanol 99.8% for HPLC, and phosphoric acid 85% m/m aqueous solution were either obtained from Thermo Fisher Scientific (Waltham, MA, USA) or from Acros Organics (Geel, Belgium). Dipotassium hydrogen phosphate 99% for analysis, tetrabutylammonium hydrogen sulphate 98%, 2-methyl-2-propanol 99.5% for analysis, and tetrahydrofuran 99.8% for HPLC were all purchased from Acros Organics (Geel, Belgium). Tetrahydrofuran for liquid chromatography without stabilizers was obtained from Merck (Darmstadt, Germany). Sodium edetate and the silica pearls with orange indicator were both obtained from Chem-lab NV (Zedelgem, Belgium) and phosphorus pentoxide was purchased from Fisher Chemical (Loughborough, UK). A Milli-Q Gradient water purification system from Millipore (Darmstadt, Germany) produced ultrapure water.

### 3.3. Sample Preparation for Irradiation Treatment

The samples of the five APIs were packed in breathable Tyvek^®^ pouches obtained from DuPont (Wilmington, DE, USA), each pouch containing 2.5 g of the API. The pouches were sealed at 130 °C with a rotary sealer type HM 782 DC-V from Hawo GmbH (Obrigheim, Germany). The dexamethasone and methylprednisolone samples, which were subjected to E-beam irradiation under non-inert and non-dried conditions, were packed in gripseal bags (10 × 15 cm, AVA NV, Temse, Belgium). The samples that needed to be sterilized under an inert atmosphere were packaged and sealed in a Tyvek^®^ pouch, and double-packed in an aluminum pouch from Long Life for Art (Eichstetten, Germany). These aluminum pouches were put under N_2_-atmosphere in a glove bag (AtmosBag size M, Sigma-Aldrich, Saint Louis, MO, USA). Before putting the aluminum pouches in the glove bag, the glove bag was flushed three times with N_2_ gas to make sure an inert atmosphere was present. Hereafter, every aluminum pouch itself was also flushed with N_2_ gas inside the glove bag to remove any potential oxygen left. When this was achieved, the Tyvek pouch was added to the aluminum pouch. To eliminate any oxygen that might enter the pouch during the packing process, and thereby maintaining an oxygen-free environment, inert samples were prepared by adding an ATCO FT100 oxygen absorber and ageless-eye oxygen indicator (both obtained from Life for Art). The oxygen indicator gives a visual indication of the oxygen inside the pouch, by being bright pink in the absence of oxygen and being purple in the presence of oxygen. In the drying experiment, to maintain the product’s moisture-free environment, dried samples were prepared in a similar manner, whereby silica pearls were inserted in the aluminum pouch to absorb any moisture inside the pouch. The APIs were dried using a Memmert U 10 oven (Memmert GmbH, Schwabach, Germany). The aluminum pouches were sealed at 160 °C with a rotary sealer type HM 782 DC-V from Hawo GmbH.

For each inert sample condition, two samples were prepared. The backup sample could be used if the first sample showed a purple oxygen indicator after the irradiation, which was never the case.

Dexamethasone and triamcinolone samples needed to be protected from light before analysis, while tetracycline hydrochloride, aciclovir, and triamcinolone samples needed to be prepared immediately before use.

### 3.4. Storage and Transport

Samples, including non-processed samples, to be processed without dry ice were stored at 2–8 °C upon receipt at the irradiation site and samples to be processed with dry ice were stored either in a −80 °C freezer or in a dry ice bath upon receipt at the irradiation site. During transport a datalogger was used to monitor the temperature.

### 3.5. Irradiation Equipment, Process, and Conditions

Gamma irradiation was carried out at the Sterigenics^®^ gamma facility in Fleurus, Belgium. E-beam irradiation of the five APIs was performed at the Sterigenics^®^ irradiation site located in Espergærde, Denmark, with a 10 MeV electron beam equipment. Different samples of each API were subjected to four distinct predetermined doses in a single pass through the irradiator (Table 8). The two highest doses of 50 kGy and 60 kGy, using E-beam irradiation, were performed in a single, as well as in a double, pass, whereby in the double pass the doses of 50 kGy and 60 kGy were split in two passes of 25 kGy and two passes of 30 kGy, respectively. Between the two passes, a time interval of 10 min was introduced to allow for cooling.

For each method and condition, non-processed samples were included as a reference and underwent no irradiation (0 kGy). They were handled the same way as the processed samples.

### 3.6. Analytical Equipment

For a clear overview, the various HPLC devices can be found in Table 9.

Chromeleon software versions 6.60 and 6.80 from Dionex were used to obtain all the chromatographic data of the Dionex system, while chromatographic data of the Hitachi Lachrom systems were acquired by HPLC software EZ Chrom Elite version 3.1.6 (Scientific Software, Inc., Pleasanton, CA, USA). The analysis of these data was performed by using Excel from Microsoft (Redmond, WA, USA) and SPSS Statistics (Versions 28.0.1.1 and 29.0.2.0, New York, NY, USA).

### 3.7. Assessment of Impurities and Relative Content by HPLC Analysis

To determine the related substances and the relative content of the five different APIs following both gamma and E-beam irradiation, the related substances test or assay described in the relevant Ph. Eur. monograph was used [17,18,19,21,22]. The applied technique was HPLC with UV/VIS detection and the related substances tests were carried out as described in the monograph, with minor adjustments to the gradient and to the composition of the mobile phases to fit the machinery and to obtain the best results. The peaks of impurities were identified in concordance with the monograph and chemical reference substance chromatograms. The chromatographic conditions that were used for the five APIs can be found in Table 10 and the gradient conditions are shown in Table 11.

To evaluate whether the API complied with the limits stated in the monograph, each impurity peak area was compared with the area of the corresponding peak in the reference solution. In addition to related substances, the content of the APIs after irradiation compared with non-processed samples was also determined.

The used chromatographic methods were all validated and if some small adjustments were needed, another method validation was performed to be certain that this adjustment still resulted in a validated HPLC analysis. When the assay of the Ph. Eur. did not include an HPLC method, a method derived from the related substances HPLC test was used.

### 3.8. Statistical Analysis

To determine the relative content of each sterilized sample, the content of the sample was expressed relative to the non-processed sample. The Mann–Whitney U test was performed as statistical analysis to assess whether there were any significant differences in content between the processed samples and the non-processed sample. The applied significance level (α) was 5% (0.05).

## 4. Conclusions

Previous studies on a set of five APIs used in ophthalmic preparations (dexamethasone, methylprednisolone, aciclovir, tetracycline hydrochloride, and triamcinolone) demonstrated that gamma irradiation under non-inert and non-dried conditions led to an increase in certain impurities in the corticosteroids, dexamethasone and methylprednisolone. Consequently, this study investigated the stability of these compounds under inert and dry conditions using gamma and E-beam irradiation, with and without the inclusion of dry ice during irradiation.

The E-beam irradiation of both dexamethasone and methylprednisolone under inert conditions did not profoundly alter the relative content of the samples. However, the formation of impurities was similar to that in gamma irradiation and was found to be reduced by the presence of dry ice for cooling the samples during irradiation, particularly at the greatest absorbed doses selected in this study, as evidenced by a reduction in impurities exceeding the limit set by the Ph. Eur. The results from the inert conditions suggest a protective effect when using N_2_ atmosphere during irradiation, especially when combined with dry ice during irradiation. Additionally, the inert conditions had a more pronounced effect on methylprednisolone compared with dexamethasone.

In contrast, pre-drying the samples before irradiation did not result in any beneficial effect on the impurity profile. Dry conditions did not yield any improvements, and caution should be exercised when making claims regarding the influence of water content during irradiation, as no drying condition was identified that preserved the original impurity profile of both steroids.

## Figures and Tables

**Table 1:** An overview of the estimated maximum acceptable dose for dexamethasone and methylprednisolone samples.

D_max,acc_ (kGy)	Non-Inert		Inert	
	Without Dry Ice	With Dry Ice	Without Dry Ice	With Dry Ice
**Dexamethasone**				
Gamma	8	10	10	13
E-beam	9	9	10	15
**Methylprednisolone**				
Gamma	NA	NA	21	40 *
E-beam	10	7	24	52 *

* Mean normalized peak area did not exceed the limit stated in the monograph [18], but the maximum acceptable dose was estimated by extrapolation. D_max,acc_, maximum acceptable dose; NA, not available.

**Table 2:** An overview of the relative contents and statistical analysis for dexamethasone and methylprednisolone samples, treated with either gamma rays or E-beam under non-inert and non-dried conditions, each processed with or without dry ice.

	Without Dry Ice			With Dry Ice		
Samples	Relative Content (%)	RSD (%)	*p*-Value	Relative Content (%)	RSD (%)	*p*-Value
**Dexamethasone (Gamma irradiation)**						
Non-processed	/	0.6	/	/	1.6	/
5 kGy	99.1	1.8	0.550	97.1	3.5	0.437
10 kGy	100.2	1.1	0.676	98.9	0.4	0.473
15 kGy	99.7	1.0	0.720	100.0	1.9	1.000
25 kGy	101.6	0.6	0.042 *	101.0	1.9	0.370
**Dexamethasone (E-beam irradiation** **)**						
Non-processed	/	0.4	/	/	0.4	/
5 kGy	102.9	0.6	0.029 *	99.3	0.4	0.057
10 kGy	101.7	0.9	0.029 *	99.2	1.7	0.486
15 kGy	100.7	0.7	0.114	98.7	0.5	0.029 *
25 kGy	99.8	0.6	0.686	99.2	0.5	0.114
**Methylprednisolone (Gamma irradiation)**						
The results on the content of methylprednisolone treated with gamma irradiation under non-inert and non-dried conditions are discussed in a previously published paper [14]. They found only a minor effect of the gamma irradiation on the relative content (98.6% to 100.5%).						
**Methylprednisolone (E-beam irradiation)**						
Non-processed	/	0.3	/	/	0.3	/
5 kGy	100.5	0.4	0.114	100.9	0.4	0.029 *
10 kGy	100.6	0.3	0.029 *	100.2	0.2	0.486
15 kGy	99.4	1.8	1.000	99.0	0.3	0.029 *
25 kGy	99.4	0.8	0.200	/	/	/

Relative content calculated vs. non-processed sample. N = 4 per condition. *p*-values significant at the 5% significance level are indicated with an asterisk (*). The observed significant differences were probably attributed to analytical errors and small RSDs. N, number of samples; RSD, relative standard deviation.

**Table 3:** An overview of the related substances test for tetracycline hydrochloride samples, treated with E-beam irradiation under non-inert and non-dried conditions, each processed with or without dry ice.

**Tetracycline Hydrochloride (Without Dry Ice)**								
**Related Substances** **Peak Area (mAU·min)**	**Non-Processed**	**25 kGy**	**35 kGy**	**50 kGy**		**60 kGy**		**Limits**
				**50 kGy**	**2 × 25 kGy**	**60 kGy**	**2 × 30 kGy**	
Impurity A (4-epitetracycline)	3.52	3.98	3.71	4.52	6.18	3.52	5.25	7.37 3.0%
Impurity B (2-acetyl-2-decarbamoyltetracycline)	1.56	1.49	1.51	1.50	1.49	1.52	1.47	3.68 1.5%
Impurity C (anhydrotetracycline)	0.96	1.01	0.93	1.03	1.10	1.04	1.12	1.89 0.5%
Impurity D (4-epianhydrotetracycline)	0.16	0.15	0.15	0.15	0.15	0.16	0.16	1.42 0.5%
**Tetracycline hydrochloride (with dry ice)**								
**Related substances**	**Non-processed**	**25 kGy**	**35 kGy**	**50 kGy**		**60 kGy**		**Limits**
				**50 kGy**	**2 × 25 kGy**	**60 kGy**	**2 × 30 kGy**	
Impurity A (4-epitetracycline)	4.66	2.95	3.48	3.20	4.06	2.79	5.04	7.37 3.0%
Impurity B (2-acetyl-2-decarbamoyltetracycline)	1.51	1.52	1.46	1.51	1.42	1.50	1.42	3.68 1.5%
Impurity C (anhydrotetracycline)	1.20	0.90	0.96	0.97	1.04	0.97	1.25	1.89 0.5%
Impurity D (4-epianhydrotetracycline)	0.16	0.16	0.16	0.16	0.15	0.16	0.17	1.42 0.5%

**Table 4:** An overview of the related substances test for aciclovir samples, treated with E-beam irradiation under non-inert and non-dried conditions, each processed with or without dry ice.

**Aciclovir (Without Dry Ice)**						
**Related Substances** **Peak Area** **(mAU·min)**	**Non-Processed**	**15 kGy**	**25 kGy**	**35 kGy**	**50 kGy**	
					**50 kGy**	**2 × 25 kGy**
Impurity B (guanine)	0.49	0.56	0.60	0.62	0.65	0.66
Limit impurity B (0.7%)	4.05	3.86	4.01	3.92	3.99	3.78
Impurity P (hydroxyethyl- guanine)	0.05 *	0.13 *	0.15 *	0.18	0.22	0.22
Limit impurity P (0.15%)	0.87	0.83	0.86	0.84	0.85	0.81
**Aciclovir (with dry ice)**						
**Related substances**	**Non-processed**	**15 kGy**	**25 kGy**	**35 kGy**	**50 kGy**	
					**50 kGy**	**2 × 25 kGy**
Impurity B (guanine)	0.51	0.56	0.60	0.63	0.62	0.67
Limit impurity B (0.7%)	4.24	4.03	4.08	4.04	3.90	4.02
Impurity P (hydroxyethyl- guanine)	0.05 *	0.10 *	0.13 *	0.17 *	0.20	0.21
Limit impurity P (0.15%)	0.91	0.86	0.87	0.87	0.84	0.86

* These mean normalized peak areas were below the disregard limit of the monograph [21].

**Table 5:** An overview of the relative contents and statistical analysis for tetracycline hydrochloride, aciclovir, and triamcinolone samples, treated with E-beam irradiation under non-inert and non-dried conditions, each processed with or without dry ice.

		Without Dry Ice			With Dry Ice		
Samples		Relative Content (%)	RSD (%)	*p*-Value	Relative Content (%)	RSD (%)	*p*-Value
**Tetracycline hydrochloride**							
Non-processed		/	0.2	/	/	0.4	/
25 kGy		99.3	1.1	0.492	100.5	1.0	0.519
35 kGy		101.1	1.4	0.367	99.6	0.6	0.465
50 kGy	50 kGy	99.7	0.1	0.323	100.5	0.2	0.414
	2 × 25 kGy	99.4	1.4	0.229	97.7	2.0	0.094
60 kGy	60 kGy	99.8	0.1	0.864	101.5	0.3	0.071
	2 × 30 kGy	99.5	0.9	0.576	98.8	0.3	0.078
**Aciclovir**							
Non-processed		/	0.9	/	/	0.9	/
15 kGy		99.5	0.7	0.617	101.4	0.9	0.317
25 kGy		102.6	0.5	0.016 *	102.4	1.8	0.147
35 kGy		100.6	0.6	0.516	99.2	0.6	0.294
50 kGy	50 kGy	99.5	3.3	0.617	98.6	2.5	0.453
	2 × 25 kGy	101.8	0.8	0.080	100.5	1.7	0.803
**Triamcinolone**							
Non-processed		/	0.3	/	/	/	/
25 kGy		100.3	0.6	0.346	/	/	/
35 kGy		98.4	1.3	0.051	98.1	/	/
50 kGy	50 kGy	100.0	0.3	0.889	99.4	/	/
	2 × 25 kGy	98.6	0.4	0.002 *	95.9	/	/
60 kGy	60 kGy	100.1	0.5	0.728	98.8	/	/
	2 × 30 kGy	99.6	0.6	0.227	97.7	/	/

Relative content calculated vs. non-processed sample. N = 4 per condition, except for triamcinolone samples processed with dry ice where N = 2 and for which statistical analysis could not be performed. *p*-values significant at the 5% significance level are indicated with an asterisk (*). N, number of samples; RSD, relative standard deviation.

**Table 6:** An overview of the relative contents and statistical analysis for dexamethasone and methylprednisolone samples, treated with gamma rays or E-beam under inert conditions, each processed with or without dry ice.

	Without Dry Ice			With Dry Ice		
Samples	Relative Content (%)	RSD (%)	*p*-Value	Relative Content (%)	RSD (%)	*p*-Value
**Dexamethasone (Gamma irradiation)**						
Non-processed	/	1.0	/	/	0.9	/
5 kGy	98.4	1.0	0.232	97.5	0.1	0.027 *
10 kGy	102.4	0.4	0.107	98.7	1.7	0.120
15 kGy	99.0	2.0	0.511	97.4	0.4	0.014 *
25 kGy	101.8	1.6	0.256	99.7	1.9	0.952
**Dexamethasone (E-beam irradiation** **)**						
Non-processed	/		/	/	1.7	/
5 kGy	101.8	2.2	0.248	102.0	1.1	0.248
10 kGy	103.6	0.6	0.021 *	103.7	0.5	0.021 *
15 kGy	97.9	0.2	0.021 *	101.0	1.1	0.248
25 kGy	98.2	1.2	0.248	99.3	1.0	0.564
**Methylprednisolone (Gamma irradiation)**						
Non-processed	/	1.3	/	/	0.6	/
5 kGy	98.2	1.5	0.248	108.0	1.2	0.021 *
10 kGy	103.1	2.2	0.043 *	101.8	0.4	0.021 *
15 kGy	95.3	0.4	0.021 *	104.1	0.8	0.021 *
25 kGy	98.5	0.8	0.083	103.8	0.7	0.021 *
**Methylprednisolone (E-beam irradiation)**						
Non-processed	/	0.9	/	/	0.1	/
5 kGy	93.2	0.6	0.021 *	102.5	0.8	0.021 *
10 kGy	96.3	1.7	0.021 *	104.8	0.4	0.021 *
15 kGy	99.1	1.7	0.386	106.0	0.8	0.021 *
25 kGy	93.6	0.4	0.021 *	101.2	0.8	0.043 *

Relative content calculated vs. non-processed sample. N = 4 per condition. *p*-values significant at the 5% significance level are indicated with an asterisk (*). N, number of samples; RSD, relative standard deviation.

**Table 7:** Effect of prior drying for 3 h on the impurities after exposure of dexamethasone and methylprednisolone samples to gamma or E-beam irradiation, each processed with or without dry ice.

Impurities After Irradiation	Without Dry Ice	With Dry Ice
**Dexamethasone (Gamma irradiation under dry conditions)**		
Impurity J	>0.15%	<0.15%
Unspecified impurity 3	>0.10%	>0.10%
**Dexamethasone (E-beam irradiation under dry conditions)**		
Impurity J	>0.15%	>0.15%
Unspecified impurity 3	>0.10%	>0.10%
**Methylprednisolone (Gamma irradiation under dry conditions)**		
Unspecified impurity 1	>0.10%	>0.10%
Unspecified impurity 3	<0.10%	>0.10%
Impurity C	>0.15%	>0.15%
**Methylprednisolone (E-beam irradiation under dry conditions)**		
Unspecified impurity 1	>0.10%	>0.10%
Unspecified impurity 3	<0.10%	>0.10%
Impurity C	>0.15%	>0.15%

**Table 8:** Target doses used to sterilize the different APIs with either gamma rays or E-beam.

Gamma irradiation	
Dexamethasone	5, 10, 15, and 25 kGy
Methylprednisolone	5, 10, 15, and 25 kGy
E-beam irradiation	
Dexamethasone	5, 10, 15, and 25 kGy
Methylprednisolone	5, 10, 15, and 25 kGy
Tetracycline hydrochloride	25, 35, 50, and 60 kGy
Aciclovir	15, 25, 35, and 50 kGy
Triamcinolone	25, 35, 50, and 60 kGy

**Table 9:** Details of HPLC equipment used for each active pharmaceutical ingredient treated with either gamma rays or E-beam irradiation.

	Dexamethasone	Methylprednisolone	Tetracycline Hydrochloride	Aciclovir	Triamcinolone
Column brand	**For all conditions** Symmetry column (4.6 × 150 mm, 5 µm particle size) from Waters (Milford, MA, USA)	**For all conditions** Inertsil ODS-3 end-capped C_18_ column (4.6 × 150 mm, 3 µm particle size) from GL Sciences (Tokyo, Japan)	**For all conditions** PLRP-S 100 Å (4.6 × 250 mm, 5 µm) from Agilent Technologies (Santa Clara, CA, USA)	**For all conditions** Supelcosil end-capped C_18_ column (4.6 × 250 mm, 5 µm particle size) from Supelco (Bellefonte, PA, USA)	**For all conditions** Alltima AQ C_18_ column (4.6 × 250 mm; 5 µm particle size) from Alltech (Kildare, Ireland)
Column heater	**E-beam** **(non-inert and non-dried conditions)** SC100 heating immersion circulator, S21P water bath from Thermo Fisher Scientific (Waltham, MA, USA) **Gamma** **(inert and dried conditions)** heated bath circulator type ED from Julabo Labortechnik GmbH **E-beam** **(inert and dried conditions)** heated bath circulator type ED from Julabo Labortechnik GmbH	**For all conditions** heated bath circulator type ED from Julabo Labortechnik GmbH (Seelbach, Germany)	**For all conditions** heated bath circulator type ED from Julabo Labortechnik GmbH	/	**/**
HPLC pump	**E-beam** **(non-inert and non-dried conditions)** Dionex P680 pump (Sunnyvale, CA, USA) **Gamma** **(inert and dried conditions)** Hitachi LaChrom Elite HPLC L-2130 pump (Tokyo, Japan) **E-beam** **(inert and dried conditions)** Merck Hitachi HPLC L-6200 Intelligent Pump (Darmstadt, Germany)	**E-beam** **(non-inert and non-dried conditions)** Hitachi LaChrom Elite HPLC L-2130 pump **Gamma and E-beam** **(inert and dried conditions)** Merck Hitachi HPLC L-6200 Intelligent Pump	**For all conditions** Merck Hitachi HPLC L-6200 Intelligent Pump	**For all conditions** Hitachi LaChrom Elite HPLC L-2130 pump	**For all conditions** Merck Hitachi HPLC L-6200 Intelligent Pump
HPLC autosampler	**E-beam** **(non-inert and non-dried conditions)** Dionex ASI-100 **Gamma** **(inert and dried conditions)** Hitachi L-2200 autosampler **E-beam** **(inert and dried conditions)** Thermo Finnigan Surveyor Autosampler Plus from Thermo Fisher Scientific (Waltham, MA, USA)	**E-beam** **(non-inert and non-dried conditions)** Hitachi L-2200 autosampler **Gamma and E-beam** **(inert and dried conditions)** Thermo Finnigan Surveyor Autosampler Plus from Thermo Fisher Scientific	**For all conditions** Thermo Finnigan Surveyor Autosampler Plus from Thermo Fisher Scientific	**For all conditions** Hitachi L-2200 autosampler	**For all conditions** Thermo Finnigan Surveyor Autosampler Plus from Thermo Fisher Scientific
HPLC UV-detector	**E-beam** **(non-inert and non-dried conditions)** Dionex UVD170U **Gamma** **(inert and dried conditions)** Hitachi L-2400 UV-detector **E-beam** **(inert and dried conditions)** Merck Hitachi L-4000 UV detector	**E-beam** **(non-inert and non-dried conditions)** Hitachi L-2400 UV-detector **Gamma and E-beam** **(inert and dried conditions)** Merck Hitachi L-4000 UV detector	**For all conditions** Merck Hitachi L-4000 UV detector	**For all conditions** Hitachi L-2400 UV-detector	**For all conditions** Merck Hitachi L-4000 UV detector

**Table 10:** The chromatographic conditions for each active pharmaceutical ingredient treated with either gamma rays or E-beam irradiation.

	**Dexamethasone**	**Methylprednisolone**	**Tetracycline Hydrochloride**	**Aciclovir**	**Triamcinolone**
Column type	End-capped C_18_ 4.6 × 150 mm, 5 µm	End-capped C_18_ 4.6 × 150 mm, 3 µm	PLRP-S 100 Å 4.6 × 250 mm, 5 µm	End-capped C_18_ 4.6 × 250 mm, 5 µm	Base-deactivated end-capped C_18_ 4.6 × 250 mm, 5 µm
Column temperature	45 °C	45 °C	60 °C	Room temperature	Room temperature
Mobile phase	**A** 25% ACN **B** 95% ACN	**A** H_3_PO_4_-THF-ACN-H_2_O (0.1:1.5:10:90 *V*/*V*/*V*/*V*) **B** H_3_PO_4_-THF-ACN-H_2_O (0.1:1.5:95:5 *V*/*V*/*V*/*V*)	(1) 80.0 g 2-methyl-2-propanol R (2) 200 mL water R (3) 100 mL of a 35 g/L solution of dipotassium hydrogen phosphate R (pH 9.0) (4) 200 mL of a 10 g/L solution of tetrabutylammonium hydrogen sulfate R (pH 9.0) (5) 10 mL of a 40 g/L solution of sodium edetate R (pH 9.0) (6) dilute to 1000.0 mL with water R	**A** ACN-phosphate buffer solution pH 3.1 (1:99 *V*/*V*) **B** ACN-phosphate buffer solution pH 2.5 (50:50 *V*/*V*)	Methanol–water (52.5:47.5 *V*/*V*)
Flow rate	1.2 mL/min	1.5 mL/min	1.0 mL/min	1.0 mL/min	1.0 mL/min
Run time	**Gamma** **(inert conditions)** 55 min **E-beam** **(normal, inert, and dried conditions)** 60 min	**E-beam** **(non-inert and non-dried conditions)** 45 min **Gamma and E-beam** **(inert and dried conditions)** 40 min	50 min	50 min	45 min
Detection wavelength	254 nm	247 nm	254 nm	254 nm	238 nm
Injection volume	20 µL	10 µL	20 µL	10 µL	20 µL
Autosampler temperature	Room temperature	Room temperature	6 °C	Room temperature	6 °C

**Table 11:** Gradient conditions for aciclovir, methylprednisolone, and dexamethasone treated with either gamma rays or E-beam irradiation.

**E-Beam Irradiation Under Non-Inert and Non-Dried Conditions**								
**Aciclovir**			**Methylprednisolone**			**Dexamethasone**		
Time (min)	A	B	Time (min)	A	B	Time (min)	A	B
0	100	0	0	83	17	0	100	0
5	100	0	14	83	17	15	100	0
27	70	30	30	52	48	40	0	100
40	70	30	35	83	17	45	100	0
45	100	0	45	83	17	60	100	0
50	100	0						
**Gamma or E-beam irradiation under inert and dried conditions**			**E-beam irradiation under normal, inert, and dried conditions**			**Gamma irradiation under inert conditions**		
**Methylprednisolone**			**Dexamethasone**			**Dexamethasone**		
Time (min)	A	B	Time (min)	A	B	Time (min)	A	B
0	81	19	0	100	0	0	100	0
13	81	19	15	100	0	15	100	0
20	44	56	45	0	100	40	0	100
40	81	19	50	100	0	45	100	0
			60	100	0	55	100	0

## Data Availability

The raw data supporting the conclusions of this article will be made available by the authors on request.

## Abbreviations

The following abbreviations are used in this manuscript:

API	active pharmaceutical ingredient
AU	absorbance unit
Dmax,acc	maximum acceptable dose
E-beam	electron beam
EMA	European medicines agency
Gy	gray
HPLC	high-performance liquid chromatography
N	number of samples
Ph. Eur.	European Pharmacopoeia
RSD	relative standard deviation
SAL	sterility assurance level
UV/VIS	ultraviolet/visible light
